# The pathological potential of ependymal cells in mild traumatic brain injury

**DOI:** 10.3389/fncel.2023.1216420

**Published:** 2023-06-16

**Authors:** Diana G. Nelles, Lili-Naz Hazrati

**Affiliations:** ^1^Department of Laboratory Medicine and Pathobiology, University of Toronto, Toronto, ON, Canada; ^2^The Hospital for Sick Children, Toronto, ON, Canada

**Keywords:** ependymal cells, mild traumatic brain injury, brain barriers, cerebrospinal fluid, DNA damage, cellular senescence, neural stem cells

## Abstract

Mild traumatic brain injury (mTBI) is a common neurological condition affecting millions of individuals worldwide. Although the pathology of mTBI is not fully understood, ependymal cells present a promising approach for studying the pathogenesis of mTBI. Previous studies have revealed that DNA damage in the form of γH2AX accumulates in ependymal cells following mTBI, with evidence of widespread cellular senescence in the brain. Ependymal ciliary dysfunction has also been observed, leading to altered cerebrospinal fluid homeostasis. Even though ependymal cells have not been extensively studied in the context of mTBI, these observations reflect the pathological potential of ependymal cells that may underlie the neuropathological and clinical presentations of mTBI. This mini review explores the molecular and structural alterations that have been reported in ependymal cells following mTBI, as well as the potential pathological mechanisms mediated by ependymal cells that may contribute to overall dysfunction of the brain post-mTBI. Specifically, we address the topics of DNA damage-induced cellular senescence, dysregulation of cerebrospinal fluid homeostasis, and the consequences of impaired ependymal cell barriers. Moreover, we highlight potential ependymal cell-based therapies for the treatment of mTBI, with a focus on neurogenesis, ependymal cell repair, and modulation of senescence signaling pathways. Further insight and research in this field will help to establish the role of ependymal cells in the pathogenesis of mTBI and may lead to improved treatments that leverage ependymal cells to target the origins of mTBI pathology.

## 1. Introduction

Traumatic brain injury (TBI) affects approximately 69 million people each year, representing one of the leading causes of disability and death worldwide (Dewan et al., 2019). Although TBI encompasses a wide range of neurological conditions and varies in severity, the majority of TBI cases are classified as mild TBI (mTBI), often referred to as concussion (Lefevre-Dognin et al., 2021). mTBI is defined as external biomechanical forces applied to the head causing acute brain injury and disruption of neurophysiological functions (Katz et al., 2015). Individuals with mTBI may experience a variety of behavioral, psychiatric, cognitive, and somatic changes that differ in overall nature, severity, and duration (Polinder et al., 2018). While most individuals only experience acute symptoms and fully recover, some individuals experience lingering symptoms reflective of post-concussive syndrome (PCS) (Broshek et al., 2015). Several epidemiological studies have shown that individuals with a past history of mTBI are at a greater risk of developing neurodegenerative diseases later in life (Gavett et al., 2010), such as Alzheimer’s disease (Ramos-Cejudo et al., 2018), Parkinson’s disease (Gardner et al., 2018), and chronic traumatic encephalopathy (McKee et al., 2016). Although the molecular mechanisms underlying the pathogenesis of mTBI are not fully understood, recent studies have shown that ependymal cells may be involved in the early stages of mTBI pathology (Schwab et al., 2019a).

Ependymal cells are a specialized type of epithelial cell in the central nervous system (CNS) that line the inner surfaces of the cerebral ventricles and the central canal of the spinal cord (Hamilton et al., 2009; Mirzadeh et al., 2017). Multiciliated ependymal cells form an interface between the cerebrospinal fluid (CSF)-filled ventricles and the underlying brain parenchyma (Tumani et al., 2017; Cousins et al., 2022). Despite being a permeable and partial barrier due to the lack of tight junctions (Johanson et al., 2008), ependymal cells are involved in maintaining CSF composition through the transport and exchange of ions, metabolites, and waste at the CSF-brain interface (Jiménez et al., 2014; Spector et al., 2015). Homeostatic control of the CSF is largely mediated by ependymal cilia, which orchestrate a synchronized beating pattern to help propel the CSF throughout the ventricular system (Siyahhan et al., 2014; Kumar et al., 2021). The dysfunction of ependymal cells and dysregulation of CSF dynamics have been implicated in several neurodegenerative diseases, such as multiple sclerosis (Hatrock et al., 2020), Huntington’s disease (Rodrigues et al., 2019), and recently, TBI (Xiong et al., 2014; Schwab et al., 2019a). Since ependymal cells form a barrier between the CSF and the brain, ependymal cell dysfunction likely compromises CSF dynamics and impairs essential neurophysiological processes (Jiménez et al., 2014). Although previous mTBI studies show evidence of astrocyte reactivity (Burda et al., 2016), neuronal dendrite degeneration (Gao and Chen, 2011), and blood-brain-barrier disruption (Munoz-Ballester et al., 2022), the pathological potential of ependymal cells has not been extensively investigated. This mini review examines the molecular alterations observed in ependymal cells following mTBI and reveals potential pathological mechanisms driven by ependymal cells. Moreover, we discuss innovative therapies that target ependymal cells to potentially treat the early stages of mTBI pathology.

## 2. Molecular alterations in ependymal cells following mTBI

### 2.1. DNA damage

At the molecular level, DNA damage has been shown to accumulate in ependymal cells following mTBI. In a study that examined post-mortem brain tissue from 38 male athletes with a past history of mTBI, DNA damage manifested in the form of γH2AX within the ependymal cell lining in addition to various non-neuronal cell types (Schwab et al., 2019a). In general, the H2A histone variant family member X (H2AX) is phosphorylated at Ser139 in response to double-stranded DNA breaks to form the biomarker γH2AX (Mah et al., 2010). In the past, γH2AX has been used as a marker of DNA damage and has been implicated in the DNA damage response pathway (Jackson and Bartek, 2009; Schwab et al., 2019b). By measuring DNA damage through γH2AX reactivity, Schwab et al. (2019a) elucidated three stages of DNA damage based on the localization and extent of γH2AX positivity. The lowest stage of DNA damage displayed γH2AX positivity exclusively within ependymal cells. Stage two displayed γH2AX positivity in ependymal cells, subependymal regions, subpial astrocytes, and peri-neuronal satellite cells in the gray matter. Stage three displayed γH2AX positivity in all the aforementioned cell types in addition to white matter oligodendrocytes. In every mTBI brain that displayed DNA damage, ependymal cells were consistently marked with γH2AX relative to other cell types (Schwab et al., 2019a). Although DNA damage can manifest in various cell types post-mTBI, the tendency of ependymal cells to accumulate and retain DNA damage may reflect an early molecular event in the pathogenesis of mTBI. However, it is unclear if γH2AX reactivity reflects aspects of mTBI disease progression, where damage and dysfunction begins at the ependymal cell lining and spreads to other cell types and brain regions (Schwab et al., 2019a). Moreover, the cascade of events leading to ependymal DNA damage after mTBI is unknown, though it is possible that mTBI induces oxidative stress and inflammation in the CSF, resulting in harmful secretions that accumulate and circulate in the CSF. Thus, ependymal cells may initially acquire DNA damage from prolonged exposure to the CSF, though these mechanisms have not yet been examined.

### 2.2. Cellular senescence

The presence of unresolved and persistent DNA damage that accumulates within a cell can trigger a state of cellular senescence (Roger et al., 2021). Cellular senescence is a phenotype marked by metabolic dysfunction, altered physiological processes, and chronic inflammation (Kumari and Jat, 2021). Senescent cells produce a plethora of pro-inflammatory factors, referred to as the senescence-associated secretory phenotype (SASP), which can spread to distant regions through paracrine signaling (Coppé et al., 2010). In the same study examining 38 male mTBI brains, several markers of cellular senescence were observed in bulk hippocampal tissue as well as specifically in ependymal cells (Schwab et al., 2019a). Following mTBI, widespread upregulation of SASP pathways and SASP-related genes was observed in the brain, with loss of the epigenetically modified histone H3K27Me3 specifically identified in ependymal cells (Schwab et al., 2019a). Although H3K27Me3 expression is associated with transcriptional repression, loss of H3K27Me3 can activate p16 and p21, both of which induce cellular senescence and upregulate SASP factors (Ito et al., 2018). Interestingly, loss of ependymal H3K27Me3 was found in areas of γH2AX positivity, suggesting that DNA damage-induced cellular senescence may occur within ependymal cells post-mTBI (Schwab et al., 2019a). However, other markers of cellular senescence observed in bulk hippocampal tissue, such as the loss of Lamin B1, have not been examined in ependymal cells (Schwab et al., 2019a).

### 2.3. Ependymal cilia

Structural alterations to ependymal cilia have also been observed following TBI events. In a mouse model of TBI, ependymal cilia was significantly decreased, resulting in a reduction of CSF flow and compromised nutrient-waste exchange processes (Xiong et al., 2014). Interestingly, the function and density of ependymal cilia was restored 30 days after injury via ependymal ciliogenesis (Xiong et al., 2014). These findings suggest that ependymal cilia critically regulate CSF dynamics, though it remains unclear if short periods of ependymal ciliary dysfunction cause permanent or long-lasting effects in the brain. In another single-cell RNA sequencing study examining the hippocampus of TBI mice, ependymal cells exhibited a significant decrease in cilium movement, leading to a reduction in CSF flow and enhanced activity of transition metal ions (Xing et al., 2022). Altered expression of ependymal genes have also been attributed to dysregulated CSF dynamics. For instance, upregulation of ependymal p11 leads to aberrant CSF circulation, disoriented ependymal planar polarity, and stress-induced depression, which is a common clinical presentation post-mTBI (Seo et al., 2021). Therefore, the repercussions of CSF dysregulation may contribute to both clinical and neuropathological presentations post-mTBI. It should be noted that ependymal cells have mainly been assessed for molecular changes and not structural ciliary changes in post-mortem human brain tissue. Consequently, future studies should attempt to characterize human ependymal cilia in the context of TBI, as outcomes may differ from those previously observed in rodents.

## 3. Potential pathological mechanisms of ependymal cells in mTBI

### 3.1. DNA damage-induced cellular senescence

The accumulation of DNA damage has been associated with widespread cellular senescence in the brain following mTBI (Schwab et al., 2019a). Although DNA damage-induced cellular senescence has not been specifically examined in ependymal cells, we hypothesize that γH2AX-positivity in ependymal cells leads to senescence post-mTBI. This hypothesis is supported by previous findings in post-mortem brain tissue where ependymal cells were consistently marked with γH2AX in mTBI brains displaying DNA damage, and where this persistent DNA damage may cause ependymal cells to transition into a senescent state and become dysfunctional. Although it is possible that mTBI impacts all cell types simultaneously, the persistence and consistency of γH2AX-positivity in ependymal cells reflects aspects of differential DNA repair mechanisms and DNA damage resiliency, relative to other cell types. The post-mitotic nature of ependymal cells and the inability of self-regeneration, especially when considering potential deficiencies in DNA damage repair mechanisms, may enhance their sensitivity to DNA damage and favor ependymal cellular senescence. In addition, the ability of cellular senescence to spread through paracrine signaling of SASP factors and positive feedback loops (Gonzalez-Meljem et al., 2018) may contribute to the widespread senescence observed post-mTBI. Therefore, a potential pathological mechanism of ependymal cells in the pathogenesis of mTBI may be the emergence and prolonged retention of DNA damage that preferentially triggers ependymal cells to become senescent. This may contribute to the spread of senescence signals throughout the brain and drive downstream signaling pathways that would not otherwise occur in neighboring cell types that efficiently repair DNA damage. A fundamental question critical to this hypothesis, however, is determining the biological significance of ependymal γH2AX. This is especially pertinent since widespread cellular senescence has been observed in both γH2AX-positive and γH2AX-negative ependymal cells post-mTBI (Schwab et al., 2019a). Alternatively, the possibility that DNA damage in surrounding cell types induces secondary DNA damage on ependymal cells is another hypothesis that could be further explored.

### 3.2. Dysregulated CSF composition

**FIGURE 1:**
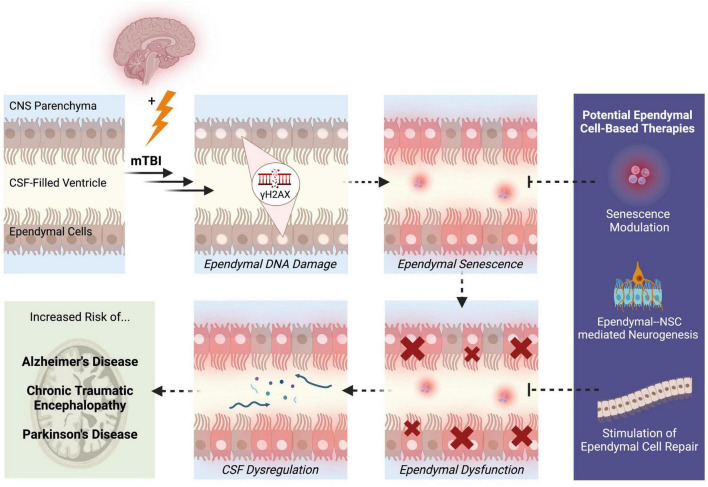
Potential pathological mechanisms of ependymal cells in the pathogenesis of mTBI. Following mTBI, ependymal cells display DNA damage in the form of γH2AX. Persistent DNA damage that is not effectively repaired through DNA repair mechanisms may trigger a state of ependymal senescence, which is accompanied by the secretion of pro-inflammatory SASP factors into the CSF. The morphological and structural changes driven by senescence may lead to ependymal cellular dysfunction and thus, impaired ependymal barrier functionality. One likely outcome of compromised ependymal barrier function is dysregulation of CSF composition, circulation, and waste-removal processes. A state of ependymal dysfunction likely increases the brain’s risk of developing further neuropathologies and disease, specifically neurodegenerative diseases like Alzheimer’s disease, chronic traumatic encephalopathy, and Parkinson’s disease. These mechanisms reveal aspects of ependymal cell pathobiology that can be targeted to treat mTBI, such as modulation of senescence signaling pathways, enhanced neurogenesis through NSC-ependymal cell crosstalk, and regeneration or repair of the ependymal cell lining. Solid arrows represent observed outcomes. Dashed arrows represent hypothesized outcomes.

One likely outcome of DNA damage-induced ependymal senescence is the secretion of pro-inflammatory SASP factors into the CSF (Schwab et al., 2019a). Since ependymal cells are in constant direct contact with the CSF, senescent ependymal cells may secrete SASP factors directly into the CSF. Consequently, the ventricular system may act as a conduit for senescent signals to circulate and spread to distant regions of the brain during CSF circulation and turnover. SASP factors that are secreted by ependymal cells may accumulate within the CSF, which could ultimately alter CSF composition and overwhelm the waste removal mechanisms that normally cleanse the CSF. In this way, the continual secretion of SASP factors into the CSF and the inability of dysfunctional ependymal cells to cleanse the CSF may contribute to the widespread cellular senescence that is observed post-mTBI. Impaired ependymal cell functionality may further contribute to dysregulated CSF composition due to leakage of molecules from the interstitial brain fluid into the CSF. Studies have shown that indeed, CSF dynamics and intracranial pressure is altered in TBI, which is accompanied by reduced ventricular waste clearance and inefficient delivery of neurohormones and nutrients within the CSF (Johanson et al., 2011). Altered CSF composition and flow has also been identified in neurological diseases linked to TBI. Pro-inflammatory cytokines in the CSF have been observed in psychiatric phenotypes like depression, which represents a common clinical presentation post-mTBI (Seo et al., 2021). Moreover, a reduction in CSF flow and turnover rate has been observed in Alzheimer’s disease, which may contribute to the hallmark phenotype of amyloid-beta plaque deposition (Li et al., 2022). Since mTBI has been associated with an increased risk of developing neurodegenerative diseases later in life, such as Alzheimer’s disease (Simon and Iliff, 2016), dysregulation of CSF homeostasis that begins in mTBI may persist or progressively worsen, potentially contributing to these associated clinical and neuropathological presentations of mTBI. Therefore, ependymal DNA damage causing cellular senescence may lead to overall dysfunction of ependymal cells through CSF dysregulation (Figure 1). These hypotheses highlight the importance of examining the pathological potential of ependymal cells to better understand the origins and disease progression patterns of mTBI.

## 4. Potential ependymal cell-based therapies

### 4.1. Neurogenesis

Ependymal cells are among the several non-neurogenic cell types that reside in the neurogenic niche of the ventricular-subventricular zone (V-SVZ), where they closely interact with neural stem cells (NSCs) (Redmond et al., 2019). A population of NSCs are located beneath the ependymal cell layer, where their apical cell processes extend past ependymal cells to access the CSF (Mirzadeh et al., 2008). Ependymal cells communicate with NSCs by secreting extracellular signaling factors and molecules into the CSF, which modulate neurogenesis through stimulation or inhibition of NSC activity (Quaresima et al., 2022). Although single-cell RNA sequencing has shown significant overlap in the transcriptomic profiles of ependymal cells and NSCs, ependymal cell mitosis and migration is not evident, suggesting a lack of stem cell-like behavior (Shah et al., 2018) and the dependency of neurogenesis on ependymal cell secretions. In a non-TBI brain, ependymal cells secrete the leucine-rich proteoglycan TSK which interacts with TGFβ, NOTCH, and WNT signaling pathways involved in neurogenesis, proliferation, and V-SVZ survival (Istiaq and Ohta, 2022). Moreover, ependymal LRP2 expression further promotes neurogenesis through negative modulation of BMPs (Gajera et al., 2010). Ependymal cells also secrete signals to suppress neurogenesis, such as CCN1, which associates with integrins to balance the pool of NSCs through inhibition of NSC self-renewal and differentiation (Wu et al., 2020). However, in a TBI brain, the homeostatic balance of ependymal growth factors and neurotrophins is perturbed, resulting in upregulated secretion of certain molecules into the CSF to promote neurogenesis at the sites of injury (Johanson et al., 2011; Bodnar et al., 2021). These neuromodulatory factors include FGF2, VEGF, NGF, EGF, GDNF, BDNF, and PACAP, all of which travel throughout the ventricular system to enhance the neurogenic capacity of the V-SVZ (Schänzer et al., 2004; Sun et al., 2009; Kernie and Parent, 2010; Mao et al., 2012; Bukovics et al., 2014; Lin et al., 2022). Therefore, a potential strategy to promote neurogenesis and cognitive recovery post-TBI could be the exogenous delivery of ependymal growth factors and neurotrophins into the CSF (Johanson et al., 2011).

### 4.2. Ependymal cell repair

Although ependymal cells are critically involved in neurogenesis, limited research has specifically examined the repair and regenerative capacity of the ependymal cell lining itself. Previous studies in adult rats revealed that structural damage to the blood-CSF-barrier was resolved within 24 h of an induced transient forebrain ischemia (Johanson et al., 2000) and within 2 weeks of a single non-penetrative blast injury (Kaur et al., 1996). This rapid recovery time is likely attributed to autocrine and paracrine signaling of the secreted ependymal-like growth factors and neuropeptides that swiftly activate repair mechanisms (Johanson et al., 2000). Interestingly, these studies showed no evidence of cellular mitosis, suggesting that cellular repair may be favored over cellular regeneration (Johanson et al., 2000). Ependymal cell repair is evident when considering ependymal cilia, where ependymal ciliogenesis led to full restoration of cilia functionality and density in mice 30 days after TBI (Xiong et al., 2014). If repair is indeed the primary mechanism of recovery, stimulation of ependymal genes involved in ependymal functionality, such as FOXJ1 and RFX transcriptional regulators, could be examined as potential ependymal cell recovery mechanisms (Jacquet et al., 2009; Choksi et al., 2014). However, the possibility of ependymal cell regeneration should not be dismissed despite limited research into this mechanism of recovery. Considering the success of NSC transplantation in regenerating other cell types in the brain following TBI (Ma et al., 2011; Pang et al., 2017), transplantation of NSCs that are reprogrammed to differentiate into ependymal cells may present a novel TBI treatment in the future.

### 4.3. Senescence-based approaches

Since the accumulation and retention of DNA damage in ependymal cells likely increases their susceptibility to becoming senescent, one therapeutic approach could be the use of senomorphic or senolytic compounds, acting to reduce SASP signaling or eliminate the senescent cells that secrete SASP factors, respectively (Gasek et al., 2021). These pharmaceutical agents may identify the degree to which senescent ependymal cell activity can be dampened and the extent to which the spread of senescent signals can be reduced. Another approach could be to target both senescence and neurogenesis, such as through exogenous supplementation with Nestin, a neuroepithelial stem cell protein associated with repair, regeneration, and senescence (Bernal and Arranz, 2018). Following injury induced by ionizing radiation, Nestin is upregulated in ependymal cells, reflecting initiation of neurogenesis in response to injury (Shi et al., 2002). However, Nestin expression is also associated with attenuated senescence in tumors (Zhang et al., 2018). Therefore, dual acting molecules like Nestin that simultaneously stimulate neurogenesis and dampen senescence could improve mTBI recovery. Though senescence has traditionally been linked with negative cellular effects, a recent study in salamanders displayed that the senescence-associated FGF-ERK signaling pathway promotes muscle dedifferentiation and limb bud regeneration (Walters et al., 2023). Therefore, harnessing specific signaling pathways in senescence may promote positive cellular outcomes relating to regeneration. While these outcomes have not been examined in the mammalian V-SVZ, leveraging the regeneration potential of senescent ependymal cells through molecular reprogramming may also enhance neurogenesis and dampen the harmful effects of senescence post-TBI.

## 5. Conclusion and future directions

Ependymal cells form essential barriers in the CNS that provide physical and biochemical protection to the brain and tightly regulate CSF homeostasis. Although ependymal cells are largely understudied in the context of neurological disease, studies have identified several molecular and structural alterations in ependymal cells post-mTBI. These observations have revealed potential pathological mechanisms of ependymal cells in mTBI pathology and prospective ways to manipulate ependymal cells as therapeutic targets. In order to develop novel ependymal cell-based therapies, it is necessary to first conduct genomic, structural, and functional studies that characterize the role of ependymal cells in health and disease. For instance, gene expression profiling may provide clarity regarding the significance of γH2AX-positivity in ependymal cells and the occurrence of cellular senescence, whereas ependymal cell imaging may identify structural and morphological changes causing dysfunction. In establishing a strong foundation of ependymal cell biology, the pathological mechanisms of ependymal cells in mTBI can be tested, thus improving the ability to develop novel ependymal cell-based therapies that treat the early stages of mTBI.

## Author contributions

DGN and L-NH conceptualized, drafted, and revised the manuscript. Both authors contributed to the article and approved the submitted version.
